# Predictive value of neutrophil‐to‐lymphocyte ratio for atrial fibrillation and stroke in type 2 diabetes mellitus: The Hong Kong Diabetes Study

**DOI:** 10.1002/edm2.397

**Published:** 2022-12-04

**Authors:** Carlin Chang, Jiandong Zhou, Oscar Hou In Chou, Justin Chan, Keith Sai Kit Leung, Teddy Tai Loy Lee, Wing Tak Wong, Abraham Ka Chung Wai, Tong Liu, Qingpeng Zhang, Sharen Lee, Gary Tse

**Affiliations:** ^1^ Department of Medicine Queen Mary Hospital Pokfulam Hong Kong China; ^2^ School of Data Science City University of Hong Kong Kowloon Hong Kong China; ^3^ Diabetes Research Unit, Cardiovascular Analytics Group UK Collaboration Hong Kong China; ^4^ Aston Medical School Aston University Birmingham UK; ^5^ Emergency Medicine Unit, Li Ka Shing Faculty of Medicine The University of Hong Kong Hong Kong China; ^6^ School of Life Sciences The Chinese University of Hong Kong Hong Kong China; ^7^ Tianjin Key Laboratory of Ionic‐Molecular Function of Cardiovascular Disease, Department of Cardiology Tianjin Institute of Cardiology, Second Hospital of Tianjin Medical University Tianjin China; ^8^ Kent and Medway Medical School Kent UK; ^9^ School of Nursing and Health Studies Hong Kong Metropolitan University Hong Kong China

**Keywords:** atrial fibrillation, neutrophil‐to‐lymphocyte ratio, stroke

## Abstract

**Introduction:**

Neutrophil‐to‐lymphocyte ratio (NLR) is a routinely available biomarker that reflects systemic inflammation. The study evaluated the predictive value of NLR for ischemic stroke and atrial fibrillation (AF) in patients with type 2 diabetes mellitus.

**Methods:**

This was a population‐based cohort study of patients with type 2 diabetes mellitus and complete blood count tests at baseline between 1 January 1st, 2009, and 31 December, 2009, at government‐funded hospitals/clinics in Hong Kong. Follow‐up was until 31 December, 2019, or death.

**Results:**

A total of 85,351 patients (age = 67.6 ± 13.2 years old, male = 48.8%, follow‐up = 3101 ± 1441 days) were included. Univariable Cox regression found that increased NLR at quartiles 2, 3 and 4 was significantly associated with higher risks of new‐onset ischemic stroke (hazard ratio [HR]: 1.28 [1.20–1.37], *p* < .001, HR: 1.41 [1.32–1.51], *p* < .001 and HR: 1.38 [1.29–1.47], *p* < .001) and AF (HR: 1.09 [1.02–1.17], *p* < .015; HR: 1.28 [1.20–1.37], *p* < .001; HR: 1.39 [1.31–1.49], *p* < .001) compared to quartile 1. On multivariable analysis, NLR remained a significant predictor of ischemic stroke risk for quartiles 2 and 3 (quartile 2: HR: 1.14 [1.05, 1.22], *p* = .001; quartile 3: HR: 1.14 [1.06, 1.23], *p* < .001) but not quartile 4 (HR: 1.08 [0.994, 1.17], *p* = .070). NLR was not predictive of AF after adjusting for confounders (quartile 2: HR: 0.966 [0.874, 1.07], *p* = .499; quartile 3: HR: 0.978 [0.884, 1.08], *p* = .661; quartile 4: HR: 1.05 [0.935, 1.16], *p* = .462).

**Conclusion:**

NLR is a significant predictor of new‐onset ischaemic stroke after adjusting for significant confounders in Chinese type 2 diabetes patients.

## Graphical abstract

NLR is a significant predictor of new onset ischaemic stroke but not AF after adjusting for significant confounders in diabetes melltius.

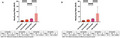

## 1 INTRODUCTION

Type 2 diabetes mellitus (T2DM) is a metabolic disease that predisposes to different adverse events, such as myocardial infarction, kidney disease, retinopathy, atrial fibrillation (AF), transient ischemic attacks and stroke.^1^, ^2^ It was estimated that 462 million people of the global population are affected by T2DM in 2017.^3^ It has an expanding prevalence and places a significant burden on healthcare systems worldwide. The chronic hyperglycaemic state could increase inflammation, such as upregulation of tumour necrosis factor‐α (TNF‐α), contributing to complications such as diabetic nephropathy.^4^ Meanwhile, chronic inflammation, especially subclinical inflammation, is thought to underlie the development of diabetic complications.^5^

Pro‐inflammatory cytokines and acute‐phase reactants, such as C‐reactive protein and interleukin‐6, are associated with the development of diabetic complications.^6^ However, cytokine profile is not routinely measured in clinical settings. White blood cell (WBC) count is a readily measured laboratory index to reflect the inflammatory status. It was recently found that the ratio between neutrophil and lymphocyte counts in the WBC has predictive values.^7^ The neutrophil‐to‐lymphocyte ratio (NLR) is a routine biomarker that reflects the inflammatory states.^8^ The balance illustrates the deleterious roles of neutrophils on endothelial function and the protective effects of lymphocytes, as the former reflects the acute/chronic inflammation while the latter reflects the adaptive immunity.^9^ Prior cross‐sectional studies found a significant relationship between NLR and increased prevalence of cardiovascular diseases, metabolic diseases, and diabetic kidney disease.^10^, ^11^, ^12^ NLR is also predictive of adverse outcomes in various conditions.^13^, ^14^, ^15^, ^16^ However, to date, there has been few detailed investigations of the possible predictive value of NLR for long‐term new‐onset AF and stroke in the context of T2DM. Therefore, we conducted this longitudinal population‐based study to determine whether NLR can be used to predict for AF and ischemic stroke among T2DM patients.

## 2 METHODS

### 2.1 Study design

This study was approved by The Joint Chinese University of Hong Kong‐New Territories East Cluster Clinical Research Ethics Committee. Patients fulfilling any of the following inclusion criteria were recruited: (1) diagnosis of T2DM using the International Classification of Disease, Ninth Edition (ICD‐9) coding system, (2) patients meeting the laboratory criteria for diabetes based on HbA1c or two successive fasting or random glucose tests as per ADA criteria, or (3) prescribed any anti‐diabetic medication between 1 January, 2009, and 31 December, 2009, at any public hospitals or outpatient clinics managed by the Hong Kong public sector, and (4) with baseline neutrophil and lymphocyte results. The data were retrieved from the Clinical Data Analysis and Reporting System (CDARS), an electronic medical database for shared comprehensive patient records across public hospitals and clinics. The system has been used for cohort studies by our team and other teams in the past,^17^, ^18^, ^19^, ^20^ including diabetes research.^21^, ^22^, ^23^

### 2.2 Data extraction

Diagnoses were obtained from ICD‐9 codes. The primary outcome was incident AF (427.31) or ischemic stroke (434–435). The code 433 (occlusion and stenosis of precerebral arteries). This is due to the very low proportion of patients coded with 433 had acute stroke.^24^ The other past comorbidities including renal, neurological comorbidities, heart failure (HF), were also extracted. Different classes of anti‐diabetic agents, antihypertensives, beta‐adrenergic receptor blocker, calcium channel blocker (CCB), diuretics and lipid‐lowering agents were also extracted. Baseline laboratory data for complete blood count (lymphocyte, neutrophil count and haemoglobin level), liver function test (alanine aminotransferase [ALT], alkaline phosphatase [ALP], albumin and total protein), renal function test (creatinine, sodium, potassium, urea), lipid (high‐density lipoprotein‐cholesterol [HDL‐C], low‐density lipoprotein‐cholesterol [LDL‐C], total cholesterol, triglyceride) and glycaemic profile (fasting blood glucose [FBG], HbA1c) between 1 January, 2008, and 31 December, 2008, were obtained. Baseline anaemia was defined as haemoglobin count <13 g/dl among male, and < 12 g/dl among female. Mean HbA1c and FBG from 1 January, 2004, and 31 December, 2008, were also calculated.

### 2.3 Statistical analysis

Statistical descriptive statistics summarized the baseline and clinical characteristics of the type‐2 diabetes patients. Continuous variables were presented as mean (95% confidence interval [CI] or standard deviation [SD]) and categorical variables were presented count (%). Univariate Cox regression was used to identify predictors for incident episodes of both AF and ischemic stroke. Hazard ratio (HR), 95% confidence interval (CI) and *p*‐Value were reported for the Cox regression. Univariate predictors with *p*‐Value <.10 were entered into a multivariate model. The Kaplan–Meier curves were plotted to reflect the outcome of stroke and AF stratified by high and low risk of NLR.To ensure the robustness of our findings, we performed the cause‐specific hazard model and Fine and Grey's subdistribution hazard model with consideration of mortality as a competing risk event. Statistical significance was defined as *p*‐Value <.05. Statistical analyses were performed using RStudio software (Version: 1.1.456) or Prism (Version: 9.0.0).

## 3 RESULTS

### 3.1 Baseline characteristics and significant predictors of new‐onset stroke

**FIGURE 1:**
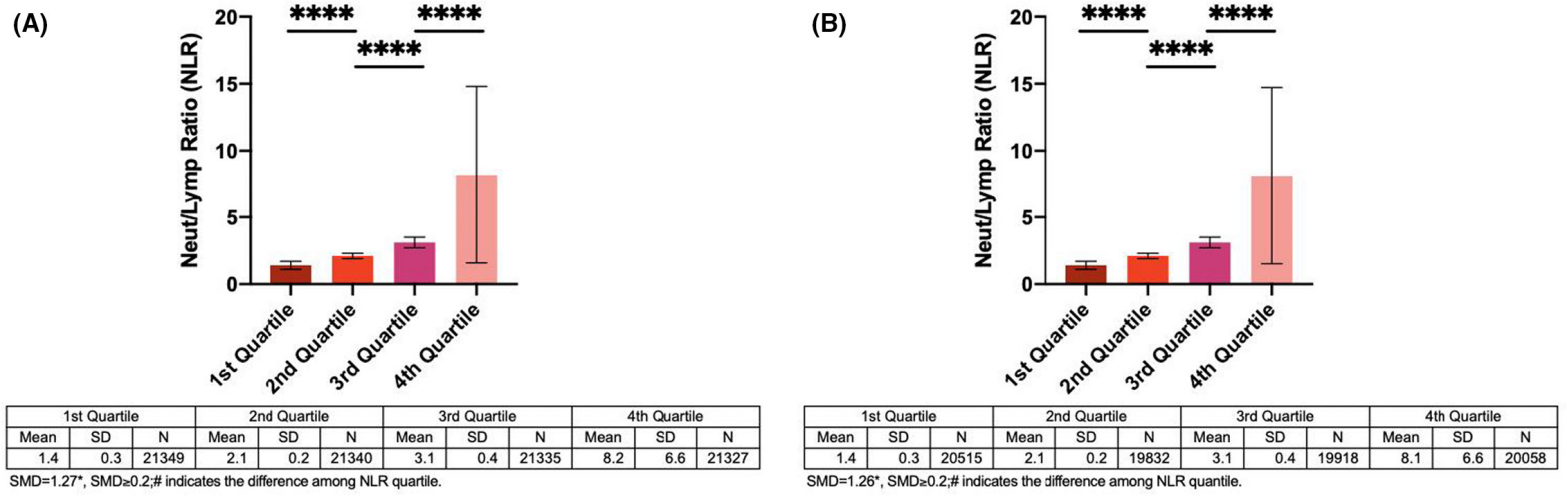
NLR of the stroke and AF cohorts stratified into quartiles (A and B) *** *p* < .0001, *** *p* < .001, ** *p* < .01, * *p* < .1.

The study cohort consisted of 85,351 patients with T2DM and neutrophil and lymphocyte values at baseline, between 1 January, 2009, and 31 December, 2009. This cohort was used for predicting the risks of new‐onset ischemic stroke (stroke cohort). This cohort was 48.8% male with a mean age of 67.6 years old (standard deviation [SD]: 13.2). During the follow‐up, 7458 patients exhibited new‐onset ischemic stroke (8.74%) and 7121 patients presented with new‐onset AF (8.87%). The baseline characteristics of the study cohort are summarized in Table 1. In addition, 80,323 patients without baseline AF were assessed separately for predicting new‐onset AF (AF cohort). The mean levels of the NLR among the stroke and AF cohorts divided into quartiles are shown in Figure 1. Those who experienced new‐onset stroke and mortality on follow‐up had significantly higher NLR values compared to the controls.

**TABLE 1:** Baseline and clinical characteristics of patients with type 2 diabetes mellitus (*n* = 85,351).

Characteristics	Mean Total (%) or Total ± SD
Age	67.6 ± 13.2
Male	41,632 (48.8)
All‐cause mortality	41,772 (48.9)
Ischemic stroke	7458 (8.74)
AF^a^	7121 (8.87)
Follow‐up duration	3101 ± 1441
NLR	3.72 ± 4.27
HDL	1.19 ± 0.353
LDL	2.84 ± 0.909
Total cholesterol	4.72 ± 1.09
Triglyceride	1.73 ± 1.46
Fasting blood glucose	7.77 ± 2.61
Biguanide	55,780 (65.4)
Sulphonylurea	56,297 (66.0)
Insulin	22,133 (25.9)
Thiazolidinedione	1790 (2.10)
DPP4	183 (0.214)
Alpha‐glucosidase inhibitor	1728 (2.02)
ACEI	46,829 (54.9)
Beta blocker	34,745 (40.7)
CCB	38,268 (44.8)
Diuretic	23,718 (27.8)
Lipid‐lowering agent	29,925 (35.1)
PVD	315 (0.369)
Ischemic stroke	7044 (8.25)
AF	5028 (5.89)
HF	8031 (9.41)
CHD	15,278 (17.9)
Hypertension	32,914 (38.6)

Abbreviation: ACEI, Angiotensin‐converting‐enzyme Inhibitors; AF, Atrial Fibrillation; CCB, Calcium Channel Blocker; CHD, Coronary Heart Disease; DPP4, Dipeptidyl‐peptidase 4; HDL, High‐density Lipoprotein; HF, Heart Failure; LDL, Low‐density Lipoprotein; NLR, Neutrophil:Lymphocyte Ratio; PVD, Peripheral vascular disease.

^a^ Prior AF excluded (*n* = 80,323).

**FIGURE 2:**
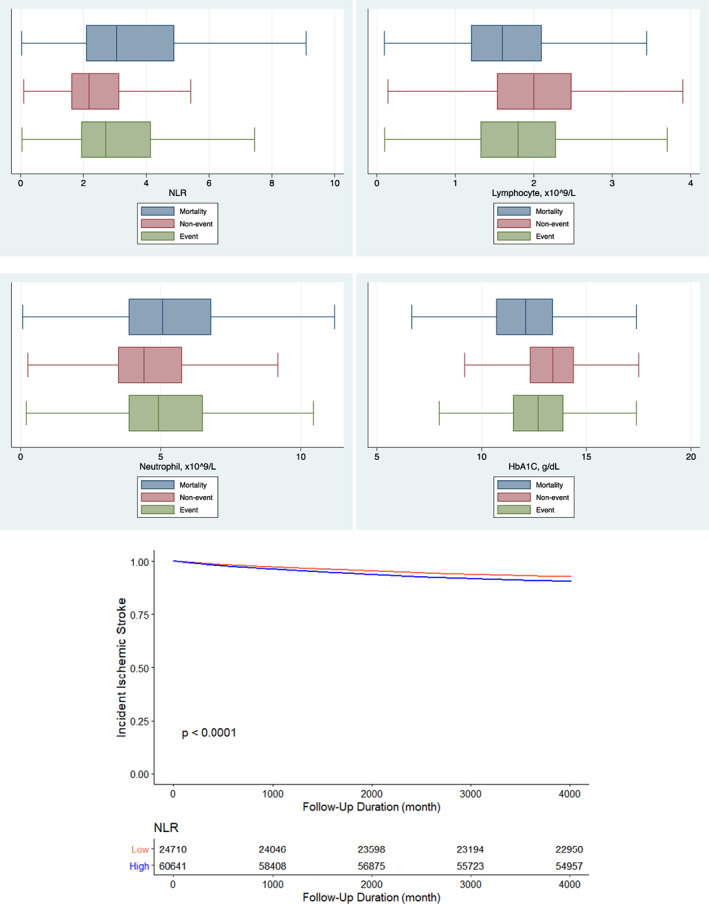
(Top Panel) Box plots for NLR, lymphocytes, neutrophils and HbA1c for patients with stroke events (blue), mortality (red), and no events (green) in the stroke cohort. (Bottom Panel) Kaplan–Meier curves for the incidence of stroke stratified by high and low levels of NLR.

In the stroke cohort, box plots for NLR, lymphocytes, neutrophils and HbA1c for patients with stroke events (blue), mortality (red) and no events (green) are shown (Figure 2, Top panel). Univariable Cox regression showed that the higher NLR was predictive of ischemic stroke (HR: 1.01 [1.00–1.01], *p* = .0315) (Table S1). Higher NLR levels at quartiles 2, 3 and 4, respectively were predictive of stroke, using quartile 1 as the reference (HR: 1.28 [1.20–1.37], *p* < .0001, HR: 1.41 [1.32–1.51], *p* < .0001 and HR: 1.38 [1.29–1.47], *p* < .0001) (Table 2). NLR remained a significant predictor of ischemic stroke risk after adjustment for quartiles 2 and 3 (quartile 2: HR: 1.14 [1.05, 1.22], *p* = .001; quartile 3: HR: 1.14 [1.06, 1.23], *p* < .001) but significance was lost for quartile 4 (HR: 1.08 [0.994, 1.17], *p* = .070). The hazard ratios for the risks of new‐onset ischemic stroke as a function of NLR levels are shown in Figure S1. The cause‐specific and subdistribution hazard models confirmed that the NLR is associated with a higher rate of new‐onset stroke (Table S2). The Kaplan–Meier curve showed that the patients with higher NLR had higher risks of ischemic stroke during the follow‐up (*p* < .0001) (Figure 2, Bottom panel).

**TABLE 2:** Hazard ratio of quartile‐stratified NLR for the prediction of unadjusted and adjusted outcomes.

	Quartile 1	Quartile 2	Quartile 3	Quartile 4
Ischemic stroke (unadjusted)	Reference	1.28 [1.20, 1.37], *p* < .0001, *n* = 42,675	1.41 [1.32, 1.51], *p* < .0001, *n* = 42,670	1.38 [1.29, 1.47], *p* < .0001, n = 42,662
Ischemic stroke (adjusted)	Reference	1.14 [1.05, 1.22], *p* = .001, *n* = 36,156	1.14 [1.06, 1.23], *p* < .001, *n* = 35,404	1.08 [0.994, 1.17], *p* = .070, *n* = 33,306
AF (unadjusted)	Reference	1.09 [1.02, 1.17], *p* = .015, *n* = 39,912	1.28 [1.20, 1.37], *p* < .0001, *n* = 39,998	1.39 [1.31, 1.49], *p* < .0001, *n* = 40,138
AF (adjusted)	Reference	0.966 [0.874, 1.07], *p* = .499, *n* = 20,536	0.978 [0.884, 1.08], *p* = .661, *n* = 19,848	1.05 [0.935, 1.16], *p* = .462, *n* = 18,464

*Note*: Adjusted to the significant multivariate predictors (demographic, comorbidity, medication).

### 3.2 Significant predictors of new‐onset AF in patients without prior AF

**FIGURE 3:**
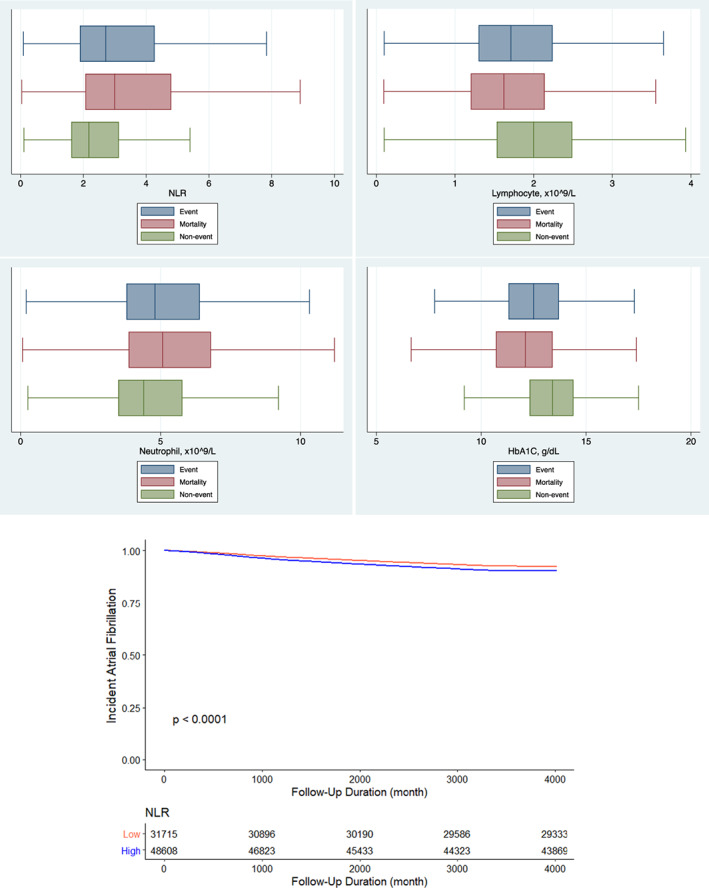
(Top Panel) Box plots for NLR, lymphocytes, neutrophils and HbA1c for patients with AF events (blue), mortality (red), and no events (green) in the atrial fibrillation (AF) cohort. (Bottom Panel) Kaplan–Meier curves for new‐onset atrial fibrillation (AF) stratified by high and low levels of NLR.

The box plots showed that patients with new‐onset AF have higher NLR values at baseline compared with those who remained in sinus rhythm (Figure 3, Top panel). Univariate Cox regression demonstrated that the NLR was predictive of AF onset (HR: 1.010 [1.005–1.015], *p* = .0001) (Table S3). Higher NLR was positively associated with higher risks of AF for quartiles 2, 3 and 4, compared with quartile 1 (HR: 1.09 [1.02–1.17], *p* < .015, HR: 1.28 [1.20–1.37], *p* < .0001 and HR: 1.39 [1.31–1.49], *p* < .0001) (Table 2). The other variables that predicted new‐onset AF identified by the univariate Cox regression are listed in Table S3. However, after adjustments, NLR was not statistically significantly in predicting the risks of AF (quartile 2: HR: 0.966 [0.874, 1.07], *p* = .499; quartile 3: HR: 0.978 [0.884, 1.08], *p* = .661; quartile 4: HR: 1.05 [0.935, 1.16], *p* = .462). Yet, the cause‐specific model and the subdistribution hazard model showed that the NLR is statistically significant in predicting the risks of new‐onset AF (Table S4). The Kaplan–Meier curve demonstrated that the patients with higher NLR have higher risks of AF during the follow‐up duration (*p* < .0001) (Figure 3, Bottom panel).

## 4 DISCUSSION

The main finding of this paper is that higher NLR was a predictor of new‐onset ischemic stroke, with a positive correlation between NLR levels and level of risk.

Inflammation contributes to the risk of stroke via a number of mechanisms, including activation of leukocytes, accumulation of pro‐inflammatory cells such as monocytes and macrophages, and platelet aggregation.^25^, ^26^ Aside from predicting new events, various haematological and inflammatory indices are also predictive of adverse outcomes following ischaemic stroke.^27^ Some are specialized tests that are available only in selected tertiary centres or for research purposes,^28^, ^29^ whilst others are based on routine blood tests and are more widely applicable.^30^, ^31^ Of the common biomarkers, the systemic inflammatory response index, calculated by the absolute neutrophil count xabsolute monocyte count/ absolute lymphocyte count, has been shown to predict poor functional outcomes despite complete or near‐complete recanalization in patients with ischaemic stroke undergoing endovascular treatment.^32^ The NLR has been shown to predict early neurological deterioration after endovascular treatment,^33^ new‐onset haemorrhagic transformation after revascularization^34^ and is associated with treatment effects for ischaemic stroke.^35^ More broadly, the NLR has been reported to be a good prognostic marker in conditions related to inflammation. Given that there are no universal standardized cut‐off for NLR, devising a stratified score would allow accounting the variations across patients with different conditions. While the neutrophil represents the activation of non‐specific inflammation, the lymphocyte represents the immune surveillance. Lymphopenia is a common finding among geriatric patients and is a significant marker of vulnerability and mortality.^36^, ^37^ The NLR has been shown to be an excellent prognostic marker for different cardiovascular diseases, including ventricular arrhythmia, HF and coronary artery disease such that higher NLR would have a negative impact on the clinical outcomes.^38^, ^39^, ^40^ Indeed, it was reported that the NLR out‐performed Framingham Risk Score model in predicting the cardiovascular‐related mortality in the general population.^41^ It also has the advantages that the differences in the referencing across laboratories would be less impacting since NLR is a ratio. Given the convenient and inexpensive nature of the NLR testing, it could be readily applied in predicting the outcome of patients with AF and ischemic stroke.

A pro‐inflammatory environment is known to play essential roles in the pathophysiology of stroke. Our data demonstrated that the NLR is predictive of incident ischaemic stroke in T2DM. Prior studies have similar reported the utility of NLR in predicting new‐onset strokes in other settings.^42^, ^43^, ^44^ Moreover, high NLR in patients with ischemic stroke have poorer functional outcomes and worse prognoses.^45^, ^46^ The neutrophil level is positively correlated with the severity of the stroke and the stroke volume.^47^ The infiltration of neutrophils into the ischaemic tissues results in the surge of inflammatory mediators and digestive enzymes.^48^ It was also shown that patients with low lymphocytes count were associated with worse outcome.^47^ Lymphocytes have a relatively controversial role in the pathophysiology of ischemia. While some studies illustrated that lymphocytes might trigger the release of pro‐inflammatory cytokines and toxins, some showed that the lymphocytes played a repairing role in ischaemic stroke.^49^, ^50^ Lymphocytes were described to increase the anti‐inflammatory cytokines, such that lymphopenia would be associated with poorer stroke protective effect.^51^ Thus, the NLR would reflect the risks of ischaemic stroke onset.

Our data showed the NLR was predictive of new‐onset AF on univariable analysis but not after adjustment for significant confounders. Nevertheless, patients who suffered from AF showed higher NLR at baseline compared to those who did not develop AF. Previous literature has found that the higher level of the NLR is predictive for the increased risks of AF occurrence or recurrence.^52^, ^53^ AF is caused by atrial fibrosis and its associated conduction disarray, which results in reentry or rapid focal ectopic firing.^54^ Inflammatory responses have an intimate relationship with AF. It was previously described that the patients with higher NLR would be associated with higher level of interleukin‐17 (IL‐17) which is involve in atrial structural remodelling.^55^ Many other inflammatory biomarkers, such as high‐sensitive C‐reactive protein (hs‐CRP) and haematological indices, are associated with higher risks of AF development.^56^, ^57^, ^58^, ^59^ In addition, Inflammation can modulate calcium homeostasis, cardiomyocytes apoptosis and fibrosis, which are contributing to the atrial structural remodelling.^60^ Last but not least, the chronic disease states among the patients with higher cardiovascular risks would further release the granulocytic myeloid‐derived suppressor cells (gMDSCs) from the bone marrow to suppress the lymphocyte release.^61^ However, upon adjustment of the other covariates, the association between NLR and AF became weaker in our results. This suggested that the NLR may also reflect the adjusted factors, including demographics and other past comorbidities, on top of directly indicating the inflammation and its effect on AF. Indeed, the NLR has been described to be associated with age, hypertension, and HF, factors that were also adjusted in our regression model.^62^, ^63^, ^64^ This indicates that NLR also reflects the other covariates, which also increased the risks of AF onset. Therefore. NLR is reflecting the specific subgroup of populations who were at higher risks of AF onset, such that clinicians are able to identify the high risks patients early and apply therapeutic agents to alleviate the condition‐related inflammations.

### 4.1 Strength and limitations

This study has several strengths. Firstly, this study is powered by a large number of patients who were followed up for a long observational period. Secondly, the territory‐wide healthcare database of Hong Kong included both outpatient clinic data from both family medicine and secondary specialist care, and hospital data. Therefore, diagnoses and attendances are recorded comprehensively in our database. Thirdly, the regression results were confirmed using competing risk analyses, including the cause‐specific and subdistribution hazard models. Lastly, the long follow‐up period allows capturing the temporal variability and long‐term outcomes.

However, several limitations should be noted. Firstly, given this is a retrospective study, there is a lack of analysis of clinical outcomes during real‐time follow‐up. Furthermore, this retrospective study can only show the association but not the causation relationship. Secondly, given the lack of coding of the common confounders such as smoking status, diet, and adherence to the prescribed medications, those critical factors were not adjusted in our models. Thirdly, Unfortunately, in this study, only ICD‐9 codes of 434 (Occlusion of cerebral arteries) and 435 (Transient cerebral ischemia). It was difficult to separate out large vessel occlusions from small vessel occlusions based on the ICD codes alone. Therefore, we did not perform subgroup analysis based on the subtypes of stroke by the TOAST classification. Future studies should be conducted to determine whether NLR can predict various stroke subtypes. Finally, this cohort is recruited from a single region such that external validation is needed to take account of the geographical heterogeneity.

## 5 CONCLUSION

Higher NLR is associated with an increased risk of ischemic stroke in a Chinese population of T2DM patients.

## AUTHOR CONTRIBUTIONS

**Carlin Chang:** Conceptualization (equal); data curation (equal); formal analysis (equal); investigation (equal); writing – original draft (equal). **Jiandong Zhou:** Data curation (equal); formal analysis (equal); software (equal); validation (equal); visualization (equal); writing – original draft (equal). **Oscar Hou In Chou:** Data curation (equal); formal analysis (equal); writing – original draft (equal); writing – review and editing (equal). **Justin Chan:** Data curation (equal); formal analysis (equal); investigation (equal); writing – original draft (equal). **Keith Sai Kit Leung:** Data curation (equal); formal analysis (equal); resources (equal); writing – original draft (equal). **Teddy Tai Loy Lee:** Conceptualization (equal); data curation (equal); formal analysis (equal); methodology (equal); writing – original draft (equal). **Wing Tak Wong:** Formal analysis (equal); funding acquisition (equal); methodology (equal); supervision (equal); writing – review and editing (equal). **Abraham Ka Chung Wai:** Data curation (equal); formal analysis (equal); methodology (equal); project administration (equal); resources (equal); software (equal); supervision (equal); writing – review and editing (equal). **Tong Liu:** Formal analysis (equal); investigation (equal); methodology (equal); project administration (equal); resources (equal); supervision (equal); visualization (equal); writing – original draft (equal); writing – review and editing (equal). **Qingpeng Zhang:** Formal analysis (equal); investigation (equal); methodology (equal); project administration (equal); resources (equal); supervision (equal); writing – review and editing (equal). **Sharen Lee:** Conceptualization (equal); data curation (equal); formal analysis (equal); investigation (equal); methodology (equal); project administration (equal); supervision (equal); writing – original draft (equal); writing – review and editing (equal).

## CONFLICT OF INTEREST

None.

## Supporting information


Appendix S1.

## Data Availability

An anonymized dataset can be obtained from the corresponding author upon reasonable request for research purposes.
