# Supplementary material for: Predictive value of neutrophil‐to‐lymphocyte ratio for atrial fibrillation and stroke in type 2 diabetes mellitus: The Hong Kong Diabetes Study
Source: Endocrinol Diabetes Metab. 2022 Dec 4;6(1):e397. doi: 10.1002/edm2.397 (PMC9836252; doi:10.1002/edm2.397)
Supplement: Supplementary file 1 — Appendix S1. [file EDM2-6-e397-s001.docx]

**Supplementary Appendix: Predictive value of neutrophil-to-lymphocyte ratio for atrial fibrillation and stroke in type 2 diabetes mellitus: a population-based cohort study**

**Supplementary Table 1.** Univariable Cox regression to identify significant predictors of mortality or new onset stroke with Akaike information criterion for the cutoffs.

AF: Atrial Fibrillation, NLR: Neutrophil-to-lymphocyte Ratio, HDL: High-density Lipoprotein, LDL: Low-density Lipoprotein, DPP4: Dipeptidyl-peptidase 4, ACEI: Angiotensin-converting-enzyme Inhibitors, CCB: Calcium Channel Blocker, PVD: Peripheral vascular disease, HF: Heart Failure, CHD: Coronary Heart Disease

| **Characteristics** | **Mortality HR [95% CI];P value** | **Cutoff** | **Stroke HR [95% CI];P value** | **Cutoff** |
| --- | --- | --- | --- | --- |
| Male gender | 1.08[1.06-1.10];<0.0001*** | - | 0.97[0.92-1.01];0.1547 | - |
| Baseline age, years | 1.074[1.073-1.075];<0.0001*** | 70.02 | 1.031[1.029-1.033];<0.0001*** | 66.05 |
| NLR | 1.033[1.032-1.034];<0.0001*** | 2.87 | 1.01[1.00-1.01];0.0315* | 1.80 |
| HDL | 0.83[0.80-0.85];<0.0001*** | 0.87 | 0.75[0.70-0.81];<0.0001*** | 1.12 |
| LDL | 0.93[0.92-0.94];<0.0001*** | 2.66 | 1.01[0.97-1.04];0.7207 | 2.14 |
| Total cholesterol | 0.91[0.90-0.92];<0.0001*** | 4.00 | 0.97[0.95-1.00];0.0262* | 4.49 |
| Triglyceride | 0.97[0.96-0.98];<0.0001*** | 1.95 | 1.01[1.00-1.03];0.1274 | 0.98 |
| Biguanide | 0.57[0.56-0.58];<0.0001*** | - | 1.02[0.98-1.07];0.3462 | - |
| Sulphonylurea | 1.26[1.23-1.28];<0.0001*** | - | 1.24[1.18-1.30];<0.0001*** | - |
| Insulin | 1.45[1.42-1.48];<0.0001*** | - | 0.98[0.93-1.04];0.5662 | - |
| Thiazolidinedione | 0.65[0.60-0.70];<0.0001*** | - | 0.67[0.55-0.81];<0.0001*** | - |
| ACEI/ARB | 1.28[1.26-1.31];<0.0001*** | - | 1.38[1.32-1.45];<0.0001*** | - |
| Beta-blocker | 1.27[1.24-1.29];<0.0001*** | - | 1.40[1.34-1.47];<0.0001*** | - |
| CCB | 1.71[1.68-1.74];<0.0001*** | - | 1.47[1.41-1.54];<0.0001*** | - |
| Diuretic | 2.13[2.09-2.17];<0.0001*** | - | 1.19[1.13-1.25];<0.0001*** | - |
| Lipid lowering agents | 1.06[1.04-1.08];<0.0001*** | - | 1.31[1.25-1.38];<0.0001*** | - |
| PVD | 2.51[2.22-2.83];<0.0001*** | - | 0.94[0.64-1.38];0.7622 | - |
| AF | 2.40[2.32-2.48];<0.0001*** | - | 2.42[2.26-2.59];<0.0001*** | - |
| HF | 3.28[3.19-3.36];<0.0001*** | - | 1.33[1.24-1.42];<0.0001*** | - |
| CHD | 1.67[1.63-1.70];<0.0001*** | - | 1.34[1.27-1.42];<0.0001*** | - |
| HT | 2.04[2.00-2.08];<0.0001*** | - | 1.76[1.68-1.84];<0.0001*** | - |
| Baseline fasting blood glucose | 1.00[1.00-1.01];0.1087 | 9.39 | 1.00[0.99-1.01];0.7564 | 10.30 |
| Previous Ischemic Stroke | 1.91[1.86-1.97];<0.0001*** | - | 3.62[3.43-3.82];<0.0001*** | - |

**Supplementary Table 2. Hazard ratio of NLR with competing risk analysis models in stroke study cohort.**

| **Cause-specific models** | Coefficient | Standard Error | t-value | p-value | [95% Confidence Interval] | | Significance |
| --- | --- | --- | --- | --- | --- | --- | --- |
| Stroke | 1.005 | .002 | 2.15 | .031 | 1.00 | 1.01 | ** |
| Mortality | 1.024 | .001 | 34.42 | <0.0001 | 1.023 | 1.026 | *** |
| **Subdistribution hazard models** |  |  |  |  |  |  |  |
| Stroke | 1.004 | .002 | 2.24 | .023 | 1.00 | 1.012 | ** |
| Mortality | 1.005 | .002 | 32.44 | .015 | 1.001 | 1.01 | ** |
| **** p<.01, ** p<.05, * p<.1* | | | | | | | |

AF: Atrial Fibrillation

**Supplementary Table 3.** Univariable Cox regression to identify significant predictors of mortality or new onset atrial fibrillation (AF) with Akaike information criterion for the cutoffs.

AF: Atrial Fibrillation, NLR: Neutrophil-to-lymphocyte Ratio, HDL: High-density Lipoprotein, LDL: Low-density Lipoprotein, DPP4: Dipeptidyl-peptidase 4, ACEI: Angiotensin-converting-enzyme Inhibitors, CCB: Calcium Channel Blocker, PVD: Peripheral vascular disease, HF: Heart Failure, CHD: Coronary Heart Disease

| **Characteristics** | **Mortality HR [95% CI];P value** | **Cutoff** | **AF HR [95% CI];P value** | **Cutoff** |
| --- | --- | --- | --- | --- |
| Male gender | 1.11[1.09-1.13];<0.0001*** | - | 0.85[0.81-0.89];<0.0001*** | - |
| Baseline age, years | 1.073[1.072-1.074];<0.0001*** | 70.04 | 1.05[1.04-1.05];<0.0001*** | 65.10 |
| NLR | 1.033[1.032-1.034];<0.0001*** | 2.87 | 1.010[1.005-1.015];<0.0001*** | 2.17 |
| HDL | 0.83[0.81-0.86];<0.0001*** | 0.85 | 0.90[0.83-0.97];0.0042** | 0.94 |
| LDL | 0.94[0.92-0.95];<0.0001*** | 2.45 | 0.91[0.88-0.94];<0.0001*** | 2.14 |
| Total cholesterol | 0.92[0.91-0.93];<0.0001*** | 4.00 | 0.88[0.85-0.90];<0.0001*** | 4.47 |
| Triglyceride | 0.98[0.97-0.99];<0.0001*** | 1.95 | 0.97[0.95-0.99];0.0036** | 2.10 |
| Biguanide | 0.57[0.56-0.58];<0.0001*** | - | 0.87[0.83-0.91];<0.0001*** | - |
| Sulphonylurea | 1.26[1.23-1.28];<0.0001*** | - | 1.14[1.08-1.20];<0.0001*** | - |
| Insulin | 1.46[1.43-1.50];<0.0001*** | - | 0.89[0.84-0.94];<0.0001*** | - |
| Thiazolidinedione | 0.65[0.60-0.71];<0.0001*** | - | 0.85[0.72-1.01];0.0637 | - |
| ACEI/ARB | 1.26[1.23-1.28];<0.0001*** | - | 1.46[1.40-1.54];<0.0001*** | - |
| Beta-blocker | 1.27[1.24-1.30];<0.0001*** | - | 1.74[1.67-1.83];<0.0001*** | - |
| CCB | 1.74[1.70-1.77];<0.0001*** | - | 1.76[1.68-1.85];<0.0001*** | - |
| Diuretic | 2.07[2.03-2.12];<0.0001*** | - | 1.90[1.81-1.99];<0.0001*** | - |
| Lipid lowering agents | 1.06[1.04-1.08];<0.0001*** | - | 1.27[1.21-1.33];<0.0001*** | - |
| PVD | 2.60[2.30-2.95];<0.0001*** | - | 0.84[0.56-1.27];0.4194 | - |
| HF | 3.27[3.17-3.37];<0.0001*** | - | 2.26[2.11-2.41];<0.0001*** | - |
| CHD | 1.60[1.56-1.64];<0.0001*** | - | 1.79[1.70-1.89];<0.0001*** | - |
| HT | 2.00[1.96-2.04];<0.0001*** | - | 1.57[1.50-1.64];<0.0001*** | - |
| Baseline fasting blood glucose | 1.00[1.00-1.01];0.1311 | 9.39 | 1.00[0.99-1.01];0.5473 | 5.22 |
| Previous Ischemic Stroke | 1.02[0.98-1.05];0.3090 | - | 1.02[0.94-1.10];0.6382 | - |

**Supplementary Table 4. Hazard ratio of NLR with competing risk analysis models in AF study cohort.**

| **Cause-specific models** | Coefficient | Standard Error | t-value | p-value | [95% Confidence Interval] | | Significance |
| --- | --- | --- | --- | --- | --- | --- | --- |
| AF | 1.01 | .002 | 4.23 | <0.0001 | 1.005 | 1.015 | *** |
| Mortality | 1.025 | .001 | 33.29 | <0.0001 | 1.023 | 1.026 | *** |
| **Subdistribution hazard models** |  |  |  |  |  |  |  |
| AF | 1.01 | .002 | 4.34 | <0.0001 | 1.004 | 1.013 | *** |
| Mortality | 1.01 | .002 | 34.99 | <0.0001 | 1.006 | 1.014 | *** |
| **** p<.01, ** p<.05, * p<.1*  AF: Atrial Fibrillation | | | | | | | |
|  | | | | | | | |

*
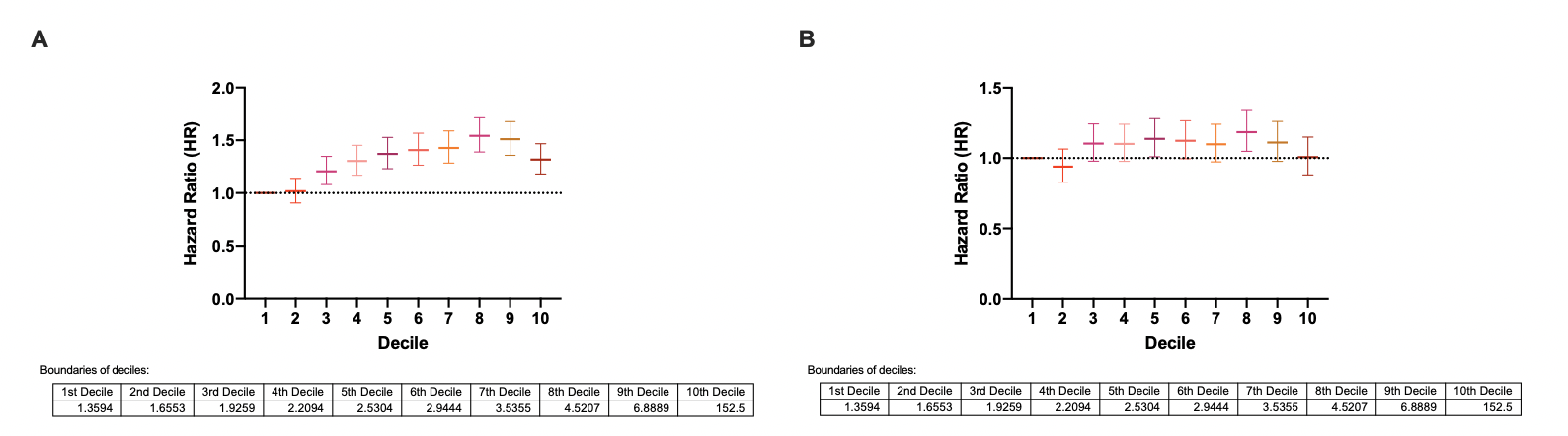
*

**Supplementary Figure 1.** The hazard ratio of stroke before (A) and after adjustments (B) as a function of NLR deciles.
